# Tilted-Beam Antenna Based on SSPPs-TL with Stable Gain

**DOI:** 10.3390/s21093288

**Published:** 2021-05-10

**Authors:** Dujuan Wei, Youlin Geng, Pengquan Zhang, Zhonghai Zhang, Chuan Yin

**Affiliations:** School of Electronics and Information, Hangzhou Dianzi University, Hangzhou 310018, China; weidujuan@hdu.edu.cn (D.W.); gengyl@hdu.edu.cn (Y.G.); zhangzhonghai@hdu.edu.cn (Z.Z.); yinc@hdu.edu.cn (C.Y.)

**Keywords:** tilted beam, SSPP structure, endfire radiation

## Abstract

In this paper, a titled-beam antenna based on spoof surface plasmon polaritons (SSPPs) transmission lines (TLs) is proposed. The parallel SSPPs-TL is a slow-wave TL, which is able to limit waves in the TL strictly. By periodically introducing a set of tapered stubs along the SSPPs-TL, the backward endfire beams are formed by the surface waves in the slow-wave radiation region. Then, through the placement of a big metal plate below the endfire antenna, the backward endfire beams are tilted, and the tilted angle of the beams are steered by the distance of the metal plate and antenna. Over the band of 5.7 GHz~7.0 GHz, the tilted antenna performs constant shapes of radiation patterns. The gain keeps stable at around 12 dBi and the 1-dB gain bandwidth is 20%. The measured results of the fabricated prototypes confirm the design theory and simulated results.

## 1. Introduction

Tilted-beam antennas have attracted tremendous attention in various applications [1,2], such as radar systems, RF base station, mobile communication systems, and satellite communication systems. These antennas are capable of radiating beams toward the intended direction to avoid the interference of noise source. Therefore, these antennas are beneficial in gaining the high signal-to-interference ratio and data rate transmission for the wireless communication systems. 

The tilted angle is a key parameter for designing a tilted-beam antenna. Generally, tilting the beams of the antenna can be accomplished by many ways, such as active beam tilting based on active components [3], mechanically tilting [4], and the passive beamsteering technique [5,6,7,8,9,10,11,12,13]. The electronically tilting beam needs the agile elements, such as diodes, phase shifter, etc., which always suffer from gain drop since a large number of components cause a large loss. With the mechanical beam tilting approach, gain is not reduced and the wide beam scanning angle is easy to obtain. However, the mechanical way has a high cost of structure and ongoing maintenance. Additionally, the larger tilted angle can lead to the increase in the height of the antenna significantly.

Based on theory of near-electric field phase transformation, a beam-tilting method is proposed [5,6] where metamaterial superstrate is stacked above the antenna within its near-field region. In [5], a metasurface superstrate is proposed that can transform the normal phase distribution of the field radiated by the antenna to a linearly increasing phase pattern, thus tilting and focusing the radiation beam. The tilted beams can also be realized by loading the metamaterial on the side of the endfire antenna [7,8,9]. EM wave radiated by the endfire antenna enters the metamaterial with a different refractive index that can be easily manipulated by the size of the unit cell, then beams are tilted with various angles. In [7], two frequency selective surface (FSS) layers with different sizes and angular rotations are used below the endfire Vivaldi antenna. After waves are reflected twice through the two FSS layers, and the endfire beams are tilted to 38°. However, the main issue of using the metamaterial is the limited frequency band of retaining the desired refractive index. Meanwhile, it also has the drawback of a bulky structure because of a large number of the periodic unit cells. Besides, a tilted beam with high gain also can be formed by arraying the radiation elements and electrically manipulating the phase of the elements [10], whereas the phase manipulation needs the electronic phase shifters or phase control circuits that increase the cost and complexity of the antenna system.

Leaky-wave antenna (LWA) with fixed tilted beams have attracted increasing interest for point-to-point or point-to-multipoint communication systems. One of the biggest problems of the fixed-beam LWA is the beam squint that leads to gain losses at the desired radiation direction. To overcome this problem, some complementary frequency-dependent metasurfaces are proposed to cancel the dispersion of the LWA [11,12,13]. In [13], two complementary dispersive prisms are proposed, one with mirror symmetry and the other one with glide symmetry, which are coupled to the LWA radiation aperture. The beam squint is reduced by the two prisms. A 11% bandwidth centered at 58 GHz is obtained with a beam squint less than ±0.9° (mirror) and ±1.7° (glide).

Spoof surface plasmon polaritons (SSPPs) have been widely studied since it was proved that the periodical subwavelength structure is able to support SPP waves in microwave and gigahertz frequencies [14]. In recent years, several designs based on SSPP waveguide with endfire radiation performances have been reported [15,16,17,18,19]. One way to obtain endfire radiation beams is by loading a main radiator and the directors at the end of an SSPPs-TL [15], the working theory of which is similar to Yagi antenna. Furthermore, another efficient method for endfire radiation is to excite the odd leaky mode of the dual-side corrugated-groove SSPP waveguide [16,17,18], to which the phase constant is well tailored by the corrugated grooves to achieve endfire radiation with the approximate satisfaction of the Hansen–Woodyard conditions. In [16], a feeding structure of a microstrip-to-slotline converter and a differential-mode exciter is used to excite the odd-mode signal on the SSPP structure. Additionally, a tapering end is introduced to the SSPP radiator, realizing a broader upper bandwidth. In [17], the TE_1_ leaky-wave mode is strategically excited by an electric slot in the center of the metal strip with dual-side corrugated grooves, which is a very simple structure exciting the odd mode. In [18], an equivalent radiating aperture is formed by gradually decreasing the grooves of an odd-mode plasmonic structure. The mechanism of SPP wave radiating out through the aperture is clarified by referring to a horn antenna.

In this paper, a fixed tilted-beam leaky-wave antenna is proposed. It is a travelling wave antenna based on the parallel SSPPs-TL. By loading a set of metal stubs, the confinement property of SSPP waves in the SSPPs-TL is broken and converted to the radiation electromagnetic waves. The proposed antenna has a phase constant for backward endfire in a wide region through the slow-wave region and the fast-wave region. Additionally, the backward endfire band is decided by the H–W conditions and the cutoff frequency of SSPPs-TL. The proposed antenna as a kind of travelling wave antenna has the potential to attain higher gain by designing a longer structure. Besides the endfire property, the prosed antenna can also realize stable tilted-upward beams over the same frequency band when a metal plate is set below the antenna. The titled beams are simply steered by the position of the metal plate. It does not need the complex feeding network and circuit system.

## 2. Antennas Design and Working Principle Analysis

### 2.1. Transmission Property Analysis of the Parallel SSPPs-TL

**Figure 1:**
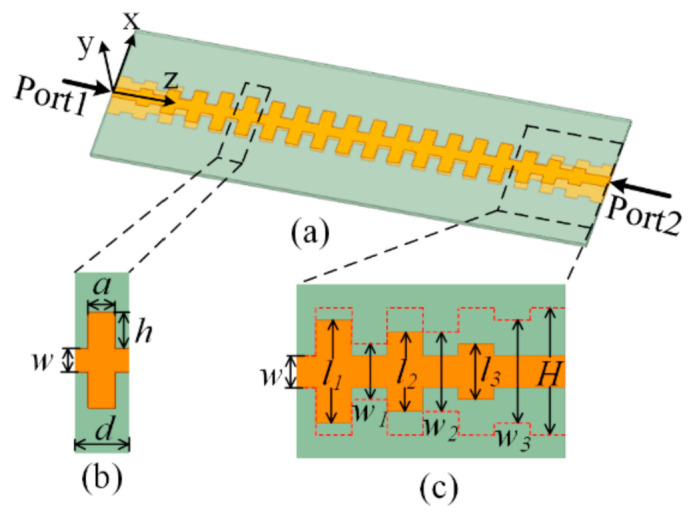
The profile of the parallel SSPPs-TL. (**a**) the full view, (**b**) a unit cell, (**c**) a transition section. *d* = 6 mm, *a* = 3 mm, *w* = 2.7 mm, *h* = 4 mm, *H* = 10.7 mm, *l*_1_ = 8.7 mm, *l*_2_ = 6.7 mm, *l*_3_ = 4.7 mm, *w*_1_ = 4.7 mm, *w*_2_ = 6.7 mm, *w*_3_ = 8.

The profile of the paralleled SSPPs-TL is shown in Figure 1. The paralleled SSPPs-TL consists of a pair of parallel striplines with corrugated grooves periodically along two sides. The period of the unit cell is d, and the height of the groove is h. The length and width of the corrugated strips are H and a. In order to realize good impedance matching between the SSPPs-TL and 50 Ω feeder, the transition section between the SSPP TL and microstrip line is designed. The top and bottom surfaces of the transition section are tapered in the opposite ways. The lengths of the corrugated strips in the bottom surface are *w*_1_, *w*_2_, and *w*_3_. The substrate F4B is utilized with dielectric constant 2.65 and thickness 1 mm. 

**Figure 2:**
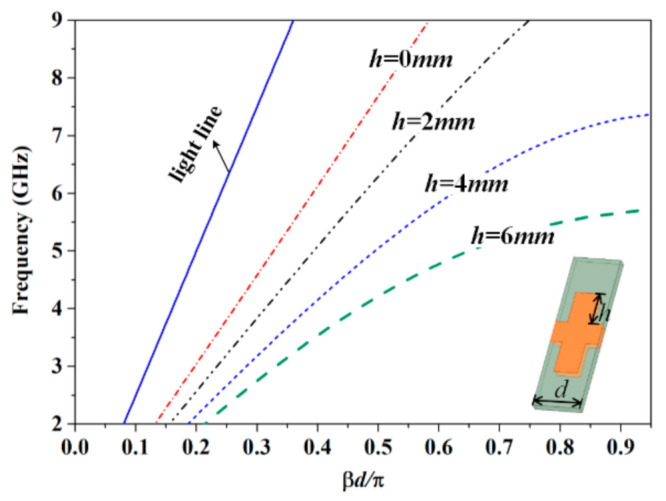
Dispersion diagram of the parallel SSPPs-TL.

The dispersion curves of the parallel SSPPs-TL with various depths of the corrugated grooves are shown in Figure 2. The SSPPs-TL support slower waves compared with the traditional slow-wave TL, which has a stronger dispersion property. As *h* is 0, the parallel SSPPs-TL becomes a balanced TL, the dispersion curve of which is a linear relationship with the frequency, approaching $k_{0}/\sqrt{\varepsilon_{r}}$. As *h* is greater than zero, the dispersion curve gradually deviates from a linearity and then the mode is prohibited, propagating at a certain frequency. With the increase in the groove depth *h*, the dispersion is stronger and stronger, and the cutoff frequency moves to a lower and lower frequency. When *h* is 4 mm and 6 mm, waves are stopped to travel through the parallel SSPPs-TL at about 7.5 GHz and 5.5 GHz, respectively. 

**Figure 3:**
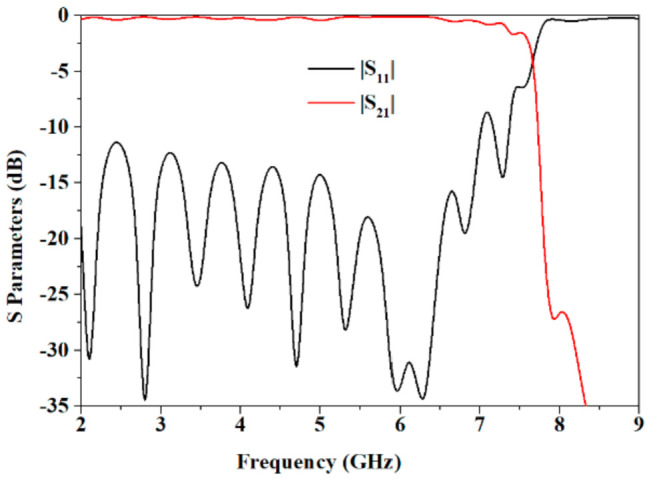
S parameters of the parallel SSPPs-TL with *h* = 4 mm.

The S parameters of the parallel SSPPs-TL with *h* = 4 mm is shown in Figure 3, which has good transmission property until 7.5 GHz.

### 2.2. Backward Endfire Radiation

**Figure 4:**
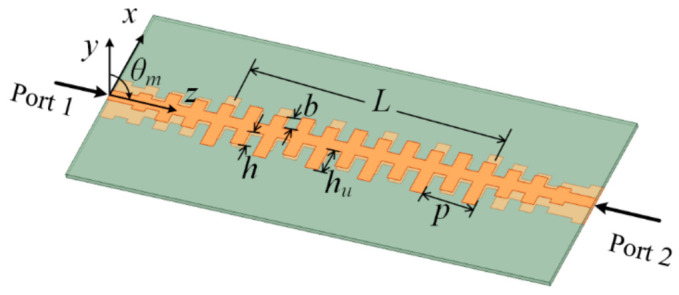
The structure of the proposed antenna with uniform stubs. *p* = 12 mm, *h* = 4 mm, *b* = 3 mm, *h_u_* = 6 mm.

**Figure 5:**
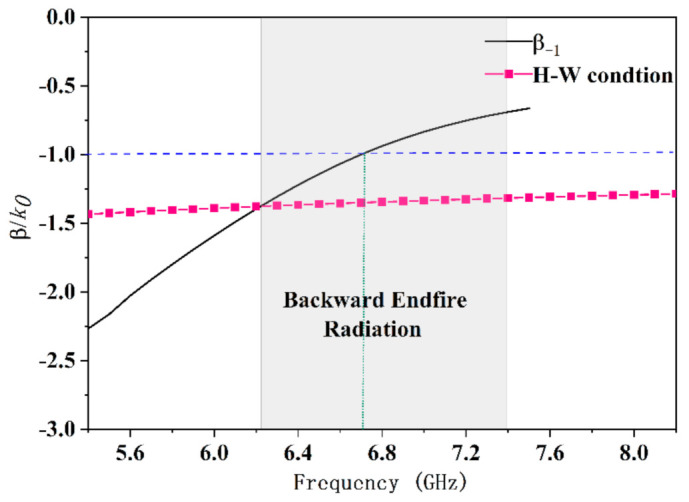
Normalized phase constant.

The parallel SSPPs-TL is a slow-wave guiding structure, as shown in Figure 2, the SSPP waves of which are confined tightly in the TL. A set of open stubs with uniform length *h_u_* are periodically introduced in the TL, as shown in Figure 4. By adjusting the groove height and the period of the unite cell, the *n* = −1 order space harmonics mode is exited. The dispersion curve of *n* = −1 mode is shown in Figure 5, and a part of the dispersion curve near the cutoff frequency is around the line of −1.

The maximum radiation angle of the linear source antenna array can be approximately determined by

(1) $$\theta_{m}≅\arcsin\left(\frac{\beta_{-1}}{k_{0}}\right)$$

where $\beta_{-1}$ is the phase constant of *n* = −1 space harmonics mode and $k_{0}$ is the propagation constant in free space. 

Based on the Hansen–Woodyard (H–W) condition, for the maximum directivity of endfire radiation, the phase constant of an antenna needs to satisfy the following equation:

(2) $$\beta \;=\left(k_{0}+\frac{2.94}{L}\right)$$

where *L* is the length of the effective radiation aperture. The normalized phase constant line of the H–W condition is also given in Figure 5.

From 6.2 GHz to 6.7 GHz, the phase constant of the antenna is between the H–W condition and the air propagation constant, so the antenna can realize backward endfire radiation based on Equations (1) and (2). From 6.7 GHz to 7.4 GHz, although the phase constant of the antenna is slightly larger than the vacuum wavenumber, the maximum radiation angle of the antenna still directs at the backward endfire direction because of superposition of dual low-elevation beams in the upper and lower spaces.

**Figure 6:**
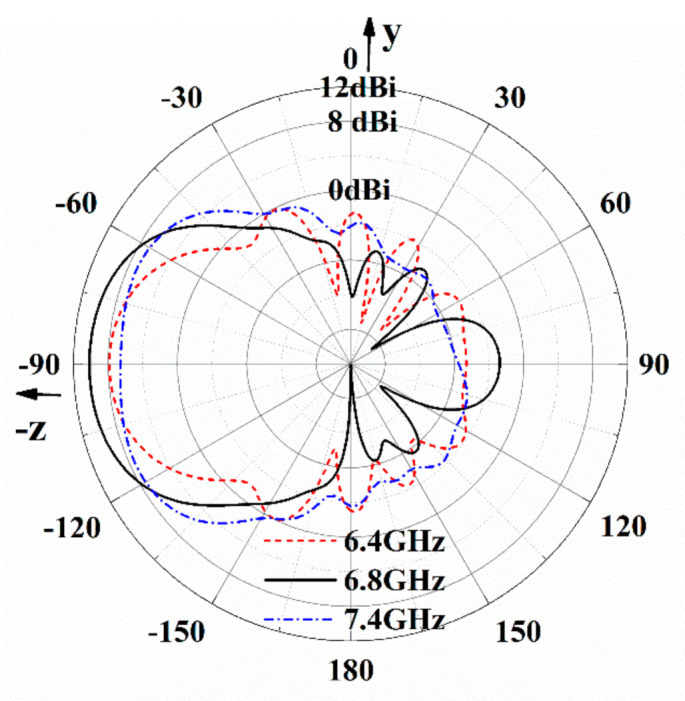
Radiation patterns of the antenna.

**Figure 7:**
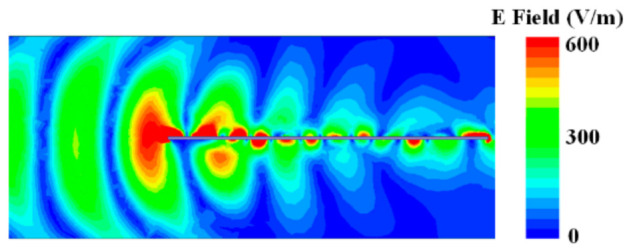
Electric field distribution of the antenna in Figure 4 at 6.6 GHz in YOZ plane.

Overall, from 6.2 GHz to 7.4 GHz, the antenna in Figure 4 has the backward endfire radiation property, and radiation patterns of the antenna given in Figure 6 have the backward endfire direction to further conform the deduction. Figure 7 gives the electric field distribution of the antenna in cross section. The electric energy mainly concentrates around the backward endfire area.

### 2.3. Stable Radiation Patterns and Titled Beams

**Figure 8:**
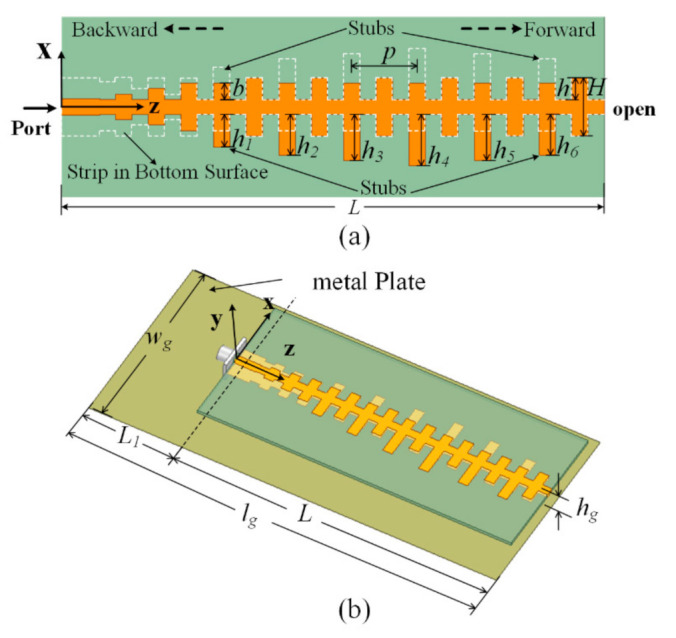
The profile of the proposed antenna. (**a**) top view, (**b**) full view. *w_g_* = 62 mm, *l_g_* = 130 mm, *L* = 100 mm, *h_g_* = 8 mm, *h*_1_ = 6 mm, *h*_2_ = 7.5 mm, *h*_3_ = 8.5 mm, *h*_4_ = 9.5 mm, *h*_5_ = 8.5 mm, *h*_6_ = 7.5 mm.

In Section 2.2, it is observed by Figure 6 that the shapes of the radiation patterns of the antenna during the working band differ obviously. In order to keep the shapes of the radiation patterns stable, the radiation stubs of the antenna are tapered. A network at one end is removed to minimize the antenna length and the end is open. The lengths of the tapered stubs are *h*_1_, *h*_2_, *h*_3_, *h*_4_, *h*_5_, and *h*_6_. Additionally, the influence of a metal plate on the antenna is also discussed in this part. The metal plate with length *l_g_* and width *w_g_* is arranged below the tapered-stub antenna with distance *h_g_*. The profile of the modified antenna is shown in Figure 8. 

**Figure 9:**
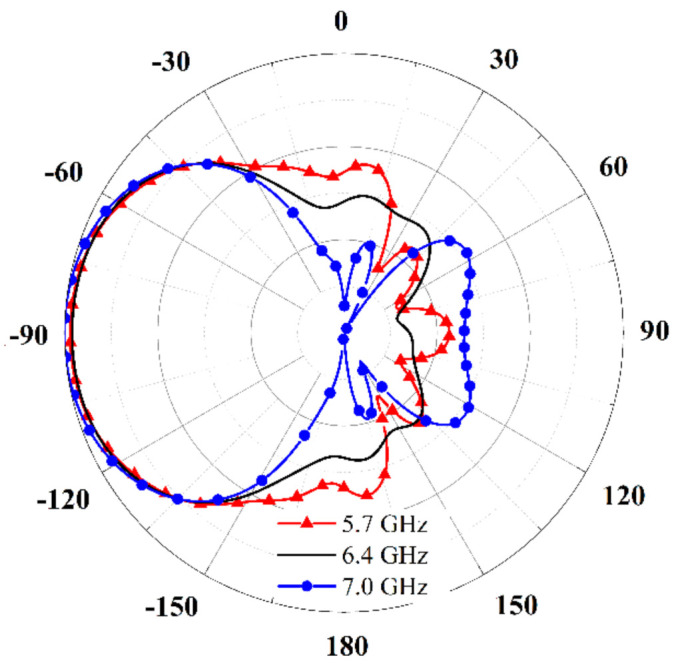
Normalized radiation patterns of the proposed antenna without metal plate in H-plane (YOZ plane).

By tapering the stubs, the radiation property of the proposed antenna obtains an obvious improvement, as shown in Figure 9. The radiation patterns become very stable from 5.7 GHz to 7.0 GHz. The realized gains are around 10 dBi, and the SLLs are less than −14 dB.

**Figure 10:**
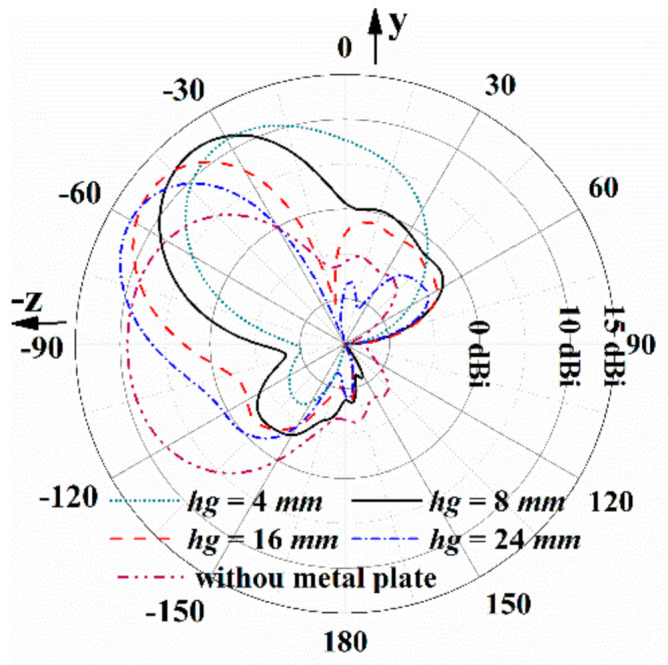
Radiation patterns of the proposed antenna with metal plate at 6.4 GHz as *h_g_* varies.

When the metal plate is set below the antenna, the backward endfire beams become tilted beams. The influence of the metal plate on the tilted angles of the radiation beams is illustrated in Figure 10. With the metal plate, the beams are tilted upward and scans from −30° to −60° with the increase in hg from 4 mm to 24 mm. The beam shape is distorted as the metal plate is too close to the antenna with *h_g_* = 4 mm. As the metal plate keeps going away from the antenna, the radiation beam approaches the backward endfire. Therefore, *h_g_* = 8 mm is chosen here to realize the fixed tilted-beam radiation, considering the low profile and the distortion of beam shape.

**Figure 11:**
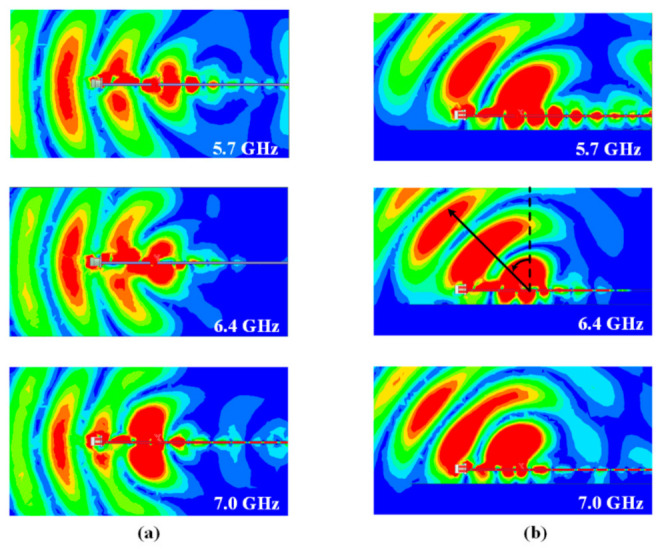
Electric field distribution of the proposed antenna in YOZ plane. (**a**) without the metal plate, (**b**) with the metal plate (*h_g_* = 8 mm).

Figure 11 gives the electric field distribution of the proposed antenna at frequency points of 5.7 GHz, 6.4 GHz, and 7.0 GHz. Without the metal plate, the electric fields at the three points all concentrate at the backward endfire direction, so the antenna radiates endfire beams, as shown in Figure 9. With the metal plate, the electric fields of the proposed antenna at the three points all concentrate at the upward tilted direction because of the reflection of the big metal plate. The strongest field is around −40°, which presents the radiation beam direction. 

The theory of beam tilted by the metal plate is analyzed below. Based on the mirror image principle, the normalized directivity function $F\left(\theta \right)$ of the proposed antenna is formed by unit factor and array factor given as:

(3) $$F\left(\theta \right)=F_{1}\left(\theta \right)\times F_{a}\left(\theta \right)$$

The unit factor $F_{1}\left(\theta \right)$ is the directivity function of the endfire antenna simulated by electromagnetic simulation software HFSS. The array factor$\;F_{a}\left(\theta \right)$ is calculated by function of *sin*(*h_g_*∙*k*_0_*cosθ*) with *h_g_ =* 8 mm, to assume the ground is infinite.

**Figure 12:**
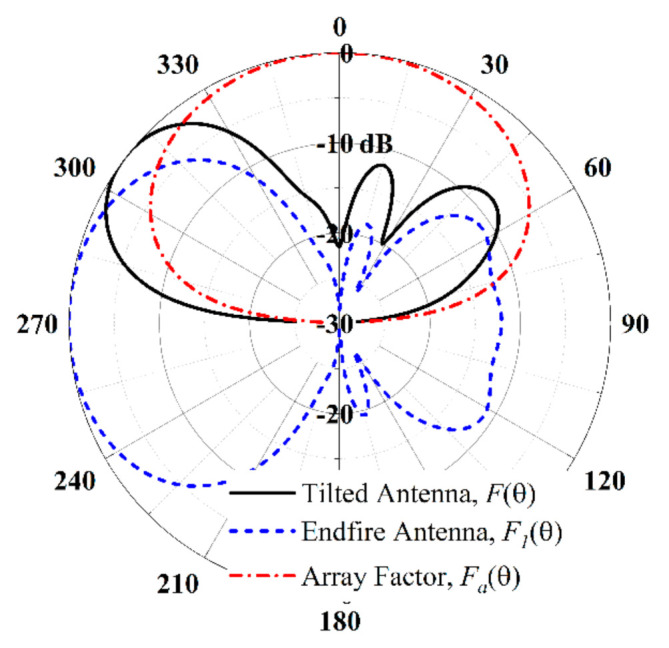
Theory of tilted-beam formation (*f* = 5.7 GHz, *h_g_* = 8 mm).

According to the function (1), the beam is upward tilted, and the calculated angle is −50°, given in Figure 12. Actually, the tilted angle of the proposed antenna with *h_g_ =* 8 mm is −40°, presented in Figure 10 and Figure 11. The difference between the angles is mainly due to the assumption of the infinite ground in the function.

**Figure 13:**
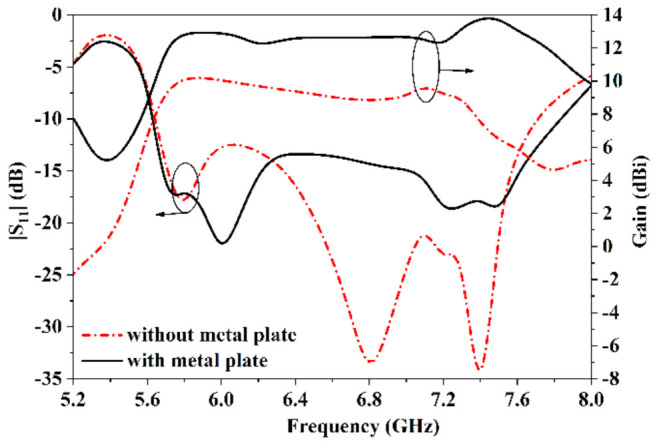
Reflective coefficients and gain of the proposed with and without plate.

Figure 13 illustrates the reflective coefficients and gains of the proposed antenna with and without the metal plate. The proposed antenna with or without the metal plate both has good impedance matching from 5.7 GHz to 7.7 GHz with |S11| < −10 dB. Without the metal plate, the proposed antenna radiates backward endfire beams with stable gain from 9.3 dBi to 10.7 dBi over the frequency band of 5.7~7.3 GHz. To introduce the metal plate, the tilted beams are formed with stable gain from 12.1 dBi to 13.8 dBi over the band of 5.7~7.7 GHz. Overall, as the metal plate is introduced, the beams of the proposed antenna are tilted, the beam-width is narrower, and the gain increases by 3 dB.

## 3. Simulated and Measured Results Discussion

**Figure 14:**
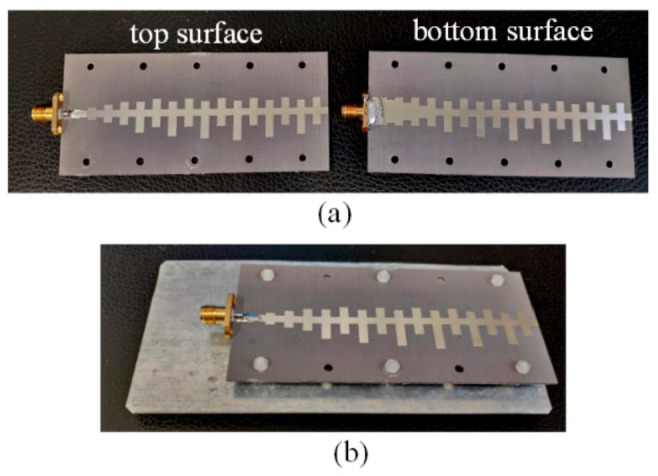
Photos of the proposed antenna. (**a**) top and bottom surfaces, (**b**) full view.

**Figure 15:**
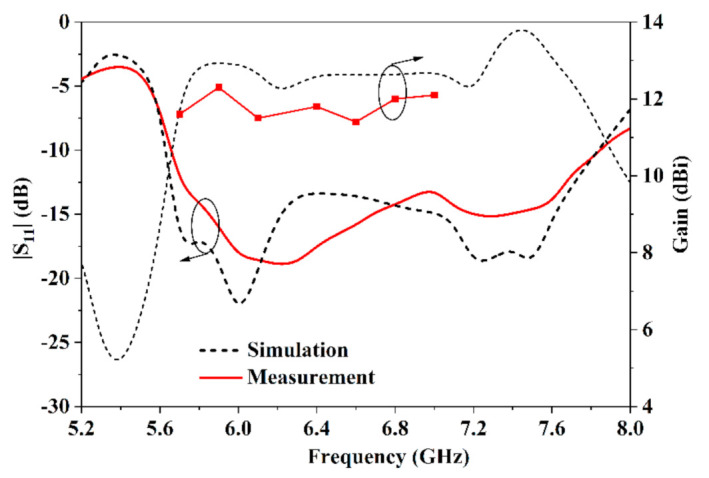
Reflective coefficients and gain of the proposed antenna.

The photo of the proposed antenna is shown in Figure 14. The distance between the metal plate and antenna is 8 mm. The reflection coefficient curve and gain of the proposed antenna is presented in Figure 15. The proposed antenna has good impedance matching from 5.7 GHz to 7.7 GHz with |S11| < −10 dB. The measured results have the same trend with the simulated results.

Over the band of 5.7~7.7 GHz, the simulated gain of the tilted antenna is from 12.1 dBi to 13.8 dBi, including 1-dB gain variation in the band of 5.7~7.0 GHz and 1.7-dB gain variation in the band of 7.0~7.7 GHz. The measured gains over the band of 5.7 ~7 GHz are slightly lower than the simulated gains from 11.6dBi~12.3dBi because of the manufactured tolerance and tested tolerance.

**Figure 16:**
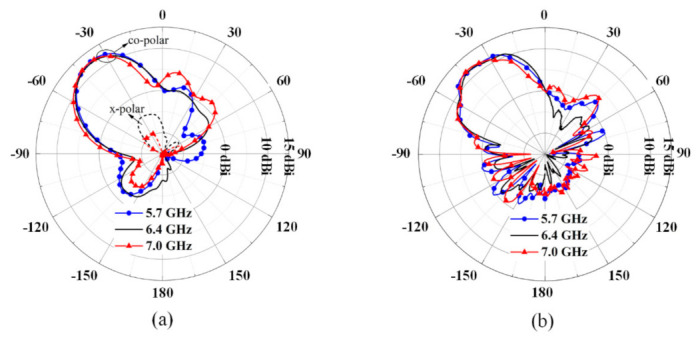
Radiation patterns of the proposed antenna in H-plane (YOZ plane). (**a**) Simulated results, (**b**) measured results.

**Figure 17:**
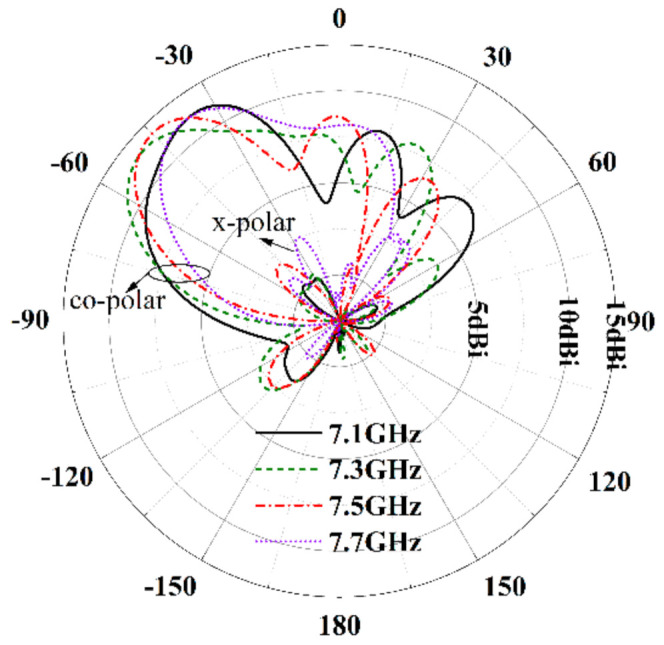
Simulated radiation patterns of the proposed antenna in YOZ plane.

The radiation patterns of the proposed antenna are illustrated in Figure 16. Over the band from 5.7 GHz to 7.0 GHz, the radiation patterns keep stable, and the beam angle is fixed at −38°. The co-polarization of the proposed antenna is along the x-axis. Additionally, the cross-polarization level is less than −17 dB. The measured results are in coincidence with the simulated results. Over the band of 7.0 GHz~7.7 GHz, the radiation beams vary from −37° to −53°, the sidelobe levels increase obviously, and the simulated gain is in the range of 11.3 dBi ~12.5 dBi at −38°, as shown in Figure 17. 

All of the previous designs in Table 1 tilt beams by loading the metamaterial plate on the antenna. These designs radiate tilted beams with various angles over a certain frequency band because of the narrow frequency band of the metamaterial retaining the desired refractive index. However, the proposed antenna can radiate fixed tilted beams over 5.7 GHz~7.0 GHz.

## 4. Conclusions

In this paper, a SSPPs-TL-based tilted-beam antenna is proposed. The backward endfire antenna consists of a set of tapered stubs periodically along the parallel SSSPPs-TL. The dispersion property of the SSPPs-TL is analyzed, which is able to confine the waves in the TL strictly. Due to the introduction of the set of tapered stubs, the continuity of the SSPPs-TL is broken, so the backward endfire beams are formed by using the surface waves in the slow-wave radiation region. Over the working frequency band from 5.7 GHz to 7.0 GHz, the shapes of endfire beams are steady. When a plane ground is arranged under the endfire antenna, the endfire beams are tilted. The tilted angle of radiation beams is steered by the distance of the antenna and the plane ground. Over the band of 5.7 GHz~7.0 GHz, the gain bandwidth of 1-dB is 20% and the gain keeps steady at around 12 dBi.

## Figures and Tables

**Table 1:** Table of performance comparison between the proposed antennas and previous works.

Ref.	Bandwidth (GHz)	Height	Gain Enhancement	Tilting Angle	Possible Tilting Rang
[5]	null	1$\lambda_{0}$	8 dB	20°	null
[7]	26 28 29	$0.37\lambda_{0}$ 2 layers	3 dB	30° 38° 36°	0~55°; 55° (4 layers)
[8]	3.4 3.5 3.6	$0.32\lambda_{0}$ 5 layers	4.7 dB 5.1 dB 5.2 dB	30° 33° 35°	Port1: −32~0°; Port2: 0–32°
[9]	5.1~5.3	$0.6\lambda_{0}$ 4 layers	0.75 dB	Port1: −38° Port2: 38°	Port1: −38~0°; Port2: 0–38°
Pro.	5.7~7.0 (20%)	0.17$\lambda_{0}$	3 dB	52°	0°~60°
