# Erratum: Wei et al. Tilted-Beam Antenna Based on SSPPs-TL with Stable Gain. *Sensors* 2021, *21*, 3288

**DOI:** 10.3390/s21186005

**Published:** 2021-09-08

**Authors:** Dujuan Wei, Youlin Geng, Pengquan Zhang, Zhonghai Zhang, Chuan Yin

**Affiliations:** School of Electronics and Information, Hangzhou Dianzi University, Hangzhou 310018, China; weidujuan@hdu.edu.cn (D.W.); gengyl@hdu.edu.cn (Y.G.); zhangzhonghai@hdu.edu.cn (Z.Z.); yinc@hdu.edu.cn (C.Y.)

**Text Correction**

There was an error in the original paper [1].

After Equation (3) on page 8, instead of “According to the function (1), the beam is upward tilted”, it should read: “According to the function (3), the beam is upward tilted”.

A correction has been made to *Section 2.3. Stable Radiation Patterns and Titled Beams,* seventh paragraph:

According to the function (3), the beam is upward tilted, and the calculated angle is −50°, given in Figure 12. Actually, the tilted angle of the proposed antenna with *h_g_* = 8 mm is −40°, presented in Figures 10 and 11. The difference between the angles is mainly due to the assumption of the infinite ground in the function.

The authors apologize for any inconvenience caused and state that the scientific conclusions are unaffected. The original article has been updated.
